# Hypothesis: can the abscopal effect explain the impact of adjuvant radiotherapy on breast cancer mortality?

**DOI:** 10.1038/s41523-018-0061-y

**Published:** 2018-04-03

**Authors:** Ismail Jatoi, John R. Benson, Ian Kunkler

**Affiliations:** 1 Department of Surgery, University of Texas Health Science Center, San Antonio, Texas USA; 2 Cambridge Breast Unit, Addenbrooke’s Hospital, Cambridge and Faculty of Medical Sciences, Anglia Ruskin University, Cambridge, UK; 3 Institute of Genetic and Molecular Medicine, Western General Hospital, University of Edinburgh, Edinburgh, UK

## Abstract

Radiotherapy is an integral component of loco-regional therapy for breast cancer. Randomized controlled trials indicate that increasing the extent of extirpative surgery primarily reduces the risk of local recurrences, while the addition of radiotherapy to surgery can also reduce the risk of distant recurrences, thereby lowering breast cancer-specific mortality. This may suggest an “abscopal” effect beyond the immediate zone of loco-regional irradiation that favorably perturbs the natural history of distant micrometastases. Immunological phenomena such as “immunogenic cell death” provide a plausible mechanistic link between the local and systemic effects of radiation. Radiotherapy treatment can stimulate both pro-immunogenic and immunosuppressive pathways with a potential net beneficial effect on anti-tumor immune activity. Upregulation of programmed cell death ligand (PD-L1) by radiotherapy is an immunosuppressive pathway that could be approached with anti-PD-L1 therapy with potential further improvement in survival. The world overview of randomized trials indicates that the breast cancer mortality reduction from adjuvant radiotherapy is delayed relative to that of adjuvant systemic treatments, and similar delays in the separation of survival curves are evident in the majority of randomized immunotherapy trials demonstrating treatment efficacy. In this article, we hypothesize that an abscopal effect may explain the benefit of radiotherapy in reducing breast cancer mortality, and that It might be possible to harness and augment this effect with systemic agents to reduce the risk of late recurrences.

## Introduction

Surgery and radiotherapy are two forms of loco-regional therapy that effectively reduce the risk of local recurrence in women with primary breast cancer.^1^ However, in contrast to surgical interventions, randomized trials have shown that addition of radiotherapy to surgery not only improves loco-regional control but can also reduce the risk of distant recurrence and death.^2–7^ Adjuvant chemotherapy seems to act synergistically to enhance the beneficial effects of radiotherapy on breast cancer mortality.^8^ Moreover, the separation of survival curves in randomized trials of adjuvant radiotherapy (i.e., radiotherapy versus no radiotherapy) is delayed when compared to trials of adjuvant systemic therapy (i.e., adjuvant systemic therapy versus no systemic therapy or comparisons of different adjuvant systemic therapy regimens).^9–11^

An explanation for the beneficial effects of radiotherapy on overall survival remains obscure. It has been postulated that local failure is a source for secondary dissemination of disease.^12^ Local failure predicts for worse survival in breast cancer patients when compared to those without locally recurrent disease after either breast-conserving surgery (BCS) or mastectomy. Thus, amongst a cohort of more than 2000 women treated with BCS and postoperative irradiation of the breast, patients with local failure had poorer 10 year survival than those maintaining local control (55% versus 75%, respectively; *P* < .001).^13^ For those patients with local recurrence, the peak time for development of disseminated disease was 5–6 years post diagnosis, whereas for patients without local recurrence (and a lower incidence of distant metastases), the corresponding time interval was only 2 years. These observations are consistent with metastatic spread from local recurrence. Nonetheless, these data do not establish a direct cause–effect relationship with several key prognostic factors being predictive for both local recurrence and survival. If a cause–effect relationship exists between local recurrence and survival, then a more modest increment in survival from postoperative radiotherapy after BCS compared to mastectomy might be attributable to the overall greater likelihood of distant recurrence secondary to chest wall recurrences after mastectomy than in- breast recurrence after BCS.

As an alternative explanation, we hypothesize in this article that breast cancer radiotherapy may have an “abscopal effect” giving rise to the beneficial effect of adjuvant radiotherapy on overall survival.^5,9^ We consider what current evidence supports this disruptive hypothesis, what challenges it, and what additional evidence would be needed to confirm it. Finally, recent advances in immunotherapy provide an opportunity to harness any abscopal effects by combining radiotherapy with immunomodulators and immune checkpoint blockade to yield clinically meaningful gains in survival outcomes.

## Biological considerations

**Fig. 1:**
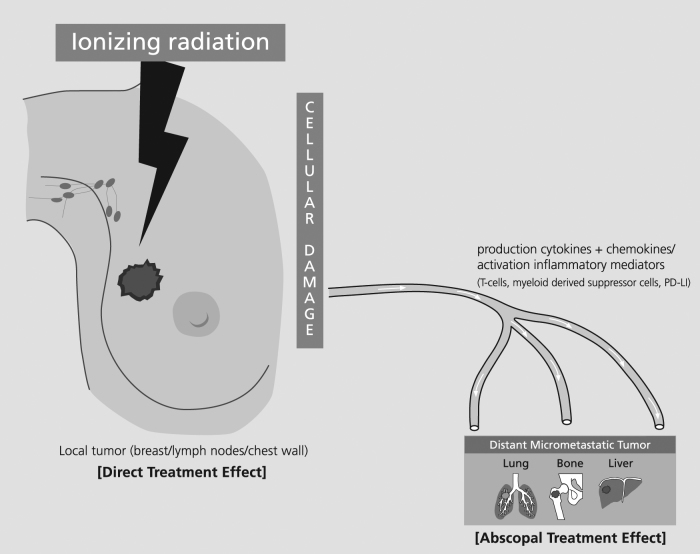
Ionizing radiation and the abscopal effect

The term “abscopal effect” was coined by R. H. Mole in 1953 as “an action at a distance from the irradiated tissue volume but within the same organism”, and alludes to radiotherapy effects at sites distant from the primary zone of irradiation (Fig. 1).^14^ Also referred to as the “distant bystander effect”, this implies that radiotherapy not only has localized action on target tissues but also out-of-field systemic anti-tumor effects.^15^

The linking of abscopal effects to the immune system was first proposed in 2004, with the observation that this effect could not occur in T cell-deficient mice.^16^ In some cases, radiotherapy may activate host immune mechanisms and immunize a patient against cancer by essentially changing the tumor into an in situ vaccine. Localized radiation may also trigger systemic immunomodulatory antitumor effects, which have been demonstrated for other tumors such as melanoma, renal cancer, and hepatocellular carcinoma.^17^ Moreover, a combination of radiotherapy with granulocyte-macrophage colony stimulating factor can generate an abscopal response amongst patients with a variety of metastatic solid tumors, including breast.^18^ The review by Reynders and colleagues identified 23 case reports and 13 preclinical studies on the abscopal effect after radiotherapy alone.^17^ Most of these effects were confined to immunogenic tumors such as melanoma and renal cell carcinoma for which a combination of immunoadjuvants with radiotherapy improved the abscopal response rates compared to radiation alone.^19^ Therapeutic strategies incorporating agents which specifically enhance host immune function may render any abscopal effects more prominent in less immunogenic tumors such as breast cancer^20^).

There is accumulating preclinical evidence supporting the idea that the clinical effectiveness of radiation involves interactions between the stroma and the tumor cell.^21^ For instance, radiotherapy may induce local cell death with subsequent release of immunogenic factors, a process termed “immunogenic cell death”.^22^ These immunogenic factors include calreticulin which is translocated to the cell surface after immunogenic cell death where processing by dendritic cells facilitates tumor antigen presentation and stimulation of cytotoxic T lymphocytes.^23,24^ Thus radiotherapy can mobilize host immune effector mechanisms which involve both pro-immunogenic and immunosuppressive effects. These might contribute to overall survival by an abscopal effect which depends upon the overall balance of these opposing influences and this in turn is determined by tissue context and radiation dosage.^25^ Furthermore, in preclinical breast cancer models, localized radiotherapy can directly stimulate release of chemokines (e.g., CXCL10 and CXCL16) that modulate immunological response by mechanisms involving increased expression of adhesion molecules such as E-selection and ICAM-1) in endothelial cells.^26^

Emerging evidence from pre-clinical models suggests that local radiation treatment can indirectly enhance systemic anti-tumor immune activity.^27,28^ Tumor-induced immune suppression not only promotes tumor growth and dissemination but also can interfere with efficiency of radiation dosage.^29^ Ideally, radiotherapy should promote immunostimulatory and minimize tumor-induced immune suppressive effects thereby leading to a net enhancement of T-cell mediated immune attack.^22,30^

One of the key immune checkpoint receptors is programmed cell death-1, which limits the response of effector T cells when bound to its programmed cell death ligands (PD-L1 and PD-L2). Radiotherapy upregulates PD-L1 in tumor cells. This in turn negatively regulates T-cell functioning and T-cell mediated anti-tumor activity, resulting in a poorer prognosis.^28,31^ The immune response of T-cells and intra-tumoral T-cell infiltration is potentiated by pharmacological blockade of the PD-L1 and PD-1 pathways, and this can increase local tumor control in melanoma and breast cancer mouse models which have been treated with a combination of radiotherapy and anti-PD-1 antibody.^27,32^ Moreover, radiotherapy combined with PD-L1 signaling blockade improves not only local control but also long-term survival compared with radiotherapy alone in tumors of syngeneic mice, further emphasizing a synergistic interaction between irradiation and anti-PD-L1 strategies.^22,30^ Likewise, this approach is supported by experiments using immunocompetent mouse models whereby blocking the PD-L1 pathway results in longer tumor growth delay following irradiation.^30^ Consistent with these observations, depletion of T-cells reduces the anti-tumor efficacy of radiation therapy which is partially compensated for by blockade of the PD-L1/PD-1 axis.^32^ Dual immune checkpoint blockade may be necessary to prevent emergence of resistance from upregulation of other checkpoint receptors.^28^

The concept of ‘radiation-induced equilibrium (RITE) has been proposed by Weichselbaum and colleagues who have evaluated the respective roles of intrinsic radiosensitivity and the immune system.^33^ These authors have explored how innate and adaptive immunity contribute to the processes of RITE and dormancy in pre-clinical models of HER2 positive breast cancer and B16 melanoma, which display heterogeneous responses to radiotherapy. Tumors that showed a variable response to radiation (cases of non-responders, early relapse, stable disease and late relapse) were excised and single cell suspensions derived from tumors. These were then irradiated ex vivo (2.5 or 10 Gy) prior to evaluation by clonogenic assays. Cells showed differential sensitivity to radiotherapy in vivo but similar radiosensitivity ex vivo. It was observed that areas of cell death were associated with a plethora of CD8+cells in stable irradiated tumors while regrowth of stable cancers was linked to a reduction in levels of CD8+cells. Therefore, RITE may represent a balance between cell death and cell regeneration which is principally mediated by CD8+cells. Furthermore, a state of therapy-induced equilibrium exists between division of tumor cells and killer cell activity by the host immune system, and this in turn determines disease status.

## Clinical implications

If radiotherapy has an abscopal effect in breast cancer patients, then why is it rarely witnessed from a clinical perspective? The relative infrequency of abscopal effects in clinical practice could be due to a number of factors. First, it is likely partly due to negation of pro-immunogenic effects of radiotherapy by counterbalancing immunosuppressive effects. Immune-based therapies can take advantage of potentially synergistic interactions between agents such as anti-PD-L1 and radiation-induced anti-tumor immune effects.^29,34^ Moreover, these systemic interactions could augment any abscopal effects and improve disease-specific survival. Crucial clinical questions relate to the timing of administration of anti-PD-L1 therapies and whether these should be concurrent with irradiation to not only maximize any abscopal effect but also harness any radiosensitizing effects of immunotherapy.^27^ Potential toxicity could ensue from simultaneous treatments, although these do appear to be more efficient in pre-clinical studies than sequential approaches.

Another possibility, yet to be proven, is that the predominant benefit of the abscopal effect is critically dependent on tumor burden. The maximal effect is perhaps on micrometastatic disease, and this might explain the beneficial effects of radiotherapy for other solid tumors in combination with systemic therapy, which may serve as the dominant partner in reducing the micrometastic cell burden. This might explain why there is minimal evidence to support our hypothesis in advanced breast cancer, with no clear evidence that loco-regional radiotherapy has any impact on distant metastases or survival outcomes in the setting of stage IV disease; in these circumstances, any abscopal effect might be overwhelmed.

To date, relatively little attention has been paid to any potential abscopal effects following breast cancer radiotherapy, but there is a growing body of evidence to support the existence of this phenomenon. Immunological (i.e., abscopal) effects can be delayed, and their potential occurrence should perhaps now be considered when designing and analyzing breast cancer radiotherapy trials.^35^ Longer follow-up of these trials might better elucidate these potential effects.^10,36^

## Evidence from clinical trials

Over the past 50 years, numerous randomized trials have compared various permutations of local therapy for breast cancer with evident differences in outcomes between trials comparing surgery alone versus those combined with radiotherapy. Randomized comparisons of surgical extent, be this in relation to the breast (breast-conserving surgery versus mastectomy), axilla (axillary surgery versus no axillary surgery or more versus less expansive axillary surgery), or the internal mammary nodes (surgical extirpation versus no extirpation), have all consistently shown that more extensive surgery reduces the risk of local recurrence, but has no corresponding reductive effect on risk of distant recurrence and mortality.^37–40^

In contrast to trials of surgical treatments, randomized trials have shown that the addition of radiotherapy to surgery not only reduces the risk of loco-regional recurrence, but can also significantly lower the risk of distant recurrence and death. Thus, both the Danish and British Columbia trials randomized high-risk breast cancer patients to modified radical mastectomy and systemic therapy versus modified radical mastectomy and systemic therapy with radiotherapy.^2,3^ The addition of radiotherapy significantly reduced the risk of distant recurrence and mortality. Furthermore, a pooled analysis of published randomized trials that compared radiotherapy versus no radiotherapy after breast conserving surgery showed that omission of radiotherapy was associated with a large increase in risk of ipsilateral breast tumor recurrence, and an increase in mortality of 8.6% (95% CI = 0.3% to 17.5%).^4^ A similar meta-analysis undertaken by the Early Breast cancer Trialists’ Collaborative Group (ECBCTCG) found that, after breast conserving surgery, radiotherapy to the conserved breast halved the rate at which the disease recurs and reduced the breast cancer death rate by about a sixth.^5^ More recently, the National Cancer Institute of Canada (NCIC) MA.20 and the European Organization for Research and Treatment of Cancer (EORTC) 22922-10925 randomized trials compared breast radiotherapy alone versus breast radiotherapy with the addition of radiotherapy to the nodal fields in patients with primary breast cancer.^6,7^ Both of these trials confirmed that extended nodal irradiation reduced not only the risk of local-regional recurrence, but also the risk of metastatic breast cancer–the latter being a surrogate for overall survival. Thus, with longer follow-up of these trials, it might be expected that addition of regional nodal radiotherapy would eventually demonstrate a survival benefit.

Results of the NCIC MA.20 and EORTC 22922-10925 trials are particularly provocative as they demonstrate a reduction in risk of distant recurrence from extended regional nodal radiotherapy, despite lack of any such benefit from more expansive surgical extirpation of regional lymph nodes. Indeed, the American College of Surgeons Oncology Group (ACOSOG) Z0011 trial which randomized sentinel node-positive breast cancer patients to sentinel node biopsy alone versus sentinel node biopsy followed by surgical clearance of the axilla, found no survival benefit for patients with sentinel node-positive tumors undergoing surgical clearance of the axilla.^39^ Moreover, amongst patients in the surgical clearance group, 27.3% had additional metastasis in non-sentinel lymph nodes, and yet leaving this residual disease behind in the group receiving sentinel node biopsy only did not affect outcomes. Thus, although both surgery and radiotherapy have traditionally been designated as “local therapies”, results of these trials raise the possibility that breast radiotherapy may also have systemic effects.

Adjuvant chest wall and breast irradiation improves overall survival significantly, whereas at present we only have evidence of a modest impact of regional nodal irradiation on disease-free survival but not overall survival in the NCIC MA-20 and EORTC 22922-10925 trials. This raises the possibility that the abscopal effect may differ between local irradiation of the breast/chest wall versus regional nodes. It is therefore not clear as to whether any potential abscopal effects become apparent only after irradiation of the primary tumor, after regional nodal irradiation as well, or whether irradiation of the primary tumor and regional nodes produce abscopal effects that are similar in magnitude.^41^ However, based on the available evidence from clinical trials, it would seem reasonable to initially explore the abscopal effect in the setting of chest wall and breast irradiation, where the overall impact of adjuvant radiotherapy is greatest.

Another important consideration is whether the abscopal response may differ according to tumor subtype. A recent analysis of a large randomized trial assessing the effect of adjuvant radiotherapy following breast-conserving surgery found that tumor subtype was not predictive of response to radiotherapy.^42^ Thus, to date, there is no evidence that tumor subtype has an influence on any potential abscopal effect.

The benefits of radiotherapy on distant recurrence and death are particularly evident in clinical trials whenever adjuvant systemic chemotherapy is administered. In the aforementioned randomized trials where an overall mortality benefit was evident following radiotherapy to either the breast or chest wall, chemotherapy was offered as adjunctive treatment. Whelan and colleagues tested a different hypothesis explaining why previous meta-analyzes had failed to demonstrate any beneficial effect of radiation therapy on mortality.^8^ These authors hypothesized that adjuvant systemic therapy was necessary for loco-regional therapy per se to manifest any mortality effect.^8^ In a meta-analysis of eighteen randomized trials where loco-regional radiotherapy was administered after surgery and all patients were treated with systemic therapy, irradiation after surgery was shown to significantly reduce overall mortality (Odds Ratio 0.83; 95% CI, 0.74 to 0.94).^8^ One might speculate that chemotherapy augments innate anti-tumor immune mechanisms by inducing immunogenic cell death and may also disrupt tumor strategies used to evade immune recognition.^43^ Alternatively, as discussed previously, adjuvant chemotherapy may lower the burden of micrometastatic disease, thereby enabling an abscopal effect. Thus, adjuvant chemotherapy may have an important role in enhancing any immunomodulatory effects of radiotherapy that can impact breast cancer mortality.

Although both systemic therapy and radiotherapy in the adjuvant setting can reduce breast cancer mortality, meta-analysis of randomized trials reveals that separation of survival curves occurs at different time-points for each modality following initial diagnosis and treatment.^9–11,36^ Thus the mortality benefit from adjuvant systemic therapy is evident within the first three years following breast cancer diagnosis and treatment, while gains from adjuvant radiotherapy emerge distinctly later (i.e., beyond three years). A delayed separation of survival curves has been observed in the majority of randomized cancer immunotherapy trials demonstrating treatment efficacy, and would be consistent with an abscopal response.^35^ Clinical trials that demonstrate a delayed response deviate from the standard proportional hazards model that is generally used in the design and analysis of cancer clinical trials.^10,36^ Under the proportional hazards model, the relative benefits of treatment are assumed to be constant over time, and delayed treatment effects might be overlooked with short-term follow-up. Extended follow-up may therefore be required to fully assess therapeutic efficacy if immunological (i.e., abscopal) effects are contributory to overall clinical outcomes.

The benefits of breast cancer radiotherapy on distant recurrence and overall mortality are generally evident in more recent trials discussed above. However, the AMAROS (After Mapping of the Axilla: Radiotherapy or Surgery} is a notable exception. This trial showed no differences in disease free survival and overall survival between the axillary radiotherapy and axillary surgery groups. Yet, the AMAROS trial was underpowered due to the small number of events, and it is therefore difficult to draw any conclusion regarding potential effects of axillary radiotherapy.

Older trials have generally failed to demonstrate a beneficial effect of radiotherapy in reducing distant recurrences and death. This might be due to infrequent or absent usage of adjuvant chemotherapy in these older trials or to outmoded radiotherapy techniques that yielded an excess of cardiac mortality. Thus, the National Surgical Adjuvant Breast and Bowel Project (NSABP)-04 and Kings/Cambridge trials pre-dated the era of modern adjuvant chemotherapy, and none of the patients were offered systemic therapy.^38,44^ Both trials failed to demonstrate any survival benefit from nodal irradiation in terms of either distant recurrence or mortality. In the NSABP-04 trial, 1079 clinically node-negative patients were randomized to mastectomy plus axillary lymph node dissection, versus mastectomy plus axillary radiotherapy, versus mastectomy and observation of the axilla with treatment intervention at relapse only.^38^ In the Kings/Cambridge trial, patients were randomized to either mastectomy plus axillary radiotherapy versus mastectomy and axillary observation.^44^ These trials suggest that surgery and radiotherapy are equally effective in reducing the risk of axillary recurrence (risk reduction approximately from 20% down to 2%), with no benefit of either axillary surgery or radiotherapy in reducing overall mortality (overall survival comparable for axillary dissection, axillary radiotherapy and observation only).

Similarly, six randomized trials were initiated more than 30 years ago comparing breast-conserving surgery (lumpectomy+radiotherapy) versus mastectomy without radiotherapy, and failed to demonstrate a benefit of breast cancer radiotherapy in lowering overall mortality (patients in both arms received axillary dissection).^45^ The largest of these breast conserving surgery trials was the NSABP-06 trial, which randomized 1851 patients to one of three arms: lumpectomy alone, lumpectomy combined with breast radiotherapy, or mastectomy.^37^ There were no differences in overall survival and no net mortality benefit from addition of radiotherapy despite a substantial effect on risk of local relapse. Nonetheless, after twenty years of follow-up, there were fewer deaths attributed to recurrent or contralateral breast cancer in the lumpectomy and radiotherapy group (198 deaths) compared with the lumpectomy alone group (242 deaths), but more non-breast cancer deaths in the lumpectomy and radiotherapy group (85 deaths) when compared to the lumpectomy group (62 deaths).^37^ It is therefore possible that radiotherapy produced an excess in non-breast cancer (i.e., cardiac) deaths amongst patients in these older trials, thereby canceling out any beneficial effects in terms of lowering breast cancer mortality. Modern tangential radiotherapy techniques avoid direct irradiation of the heart (incorporate ‘heart spare’) and deliver a more effective dose using intensity modulation to target the zone of the breast where risk of breast cancer recurrence is greatest.^46^ Thus, in the modern era, any reduction in breast cancer deaths attributable to radiotherapy can translate to improvements in overall survival.

## Additional evidence required

So what additional evidence might be needed to confirm a clinically relevant abscopal effect for adjuvant radiotherapy in early breast cancer? The current state of knowledge derived from preclinical and clinical data is incomplete and inadequate to confidently declare or refute any abscopal effect.

Both clinical and experimental data suggest that any abscopal effect occurs following adequate activation of an anti-tumor response from local irradiation.^16^ This prompted the hypothesis that combining immunotherapy with radiation could potentially be synergistic and result in enhanced immune-mediated systemic anti- tumor effects^47^

Clinically overt cancers have usually acquired resistance to immune-mediated anti-tumor effects. Immunosuppressive effects of tumors include a number of local and systemic processes such as overexpression of T cell inhibitory signals, underexpression of co-stimulatory signals and reducing presentation of antigens.^48^ A principal focus of clinical and translational studies combining immunotherapy with radiotherapy is enhancement of immunostimulatory signals while blocking inhibitory signals. Radiation in combination with immunostimulatory molecules such as Interleukin-2 (IL2), Toll-like receptor (TL-R) ligands and FMS-like tyrosine-kinase 3 (Flt-3) ligand have been used pre-clinically to engender the abscopal effect in mouse models of breast cancer. In one study, breast cancer cells were implanted bilaterally into the flanks of mice, one side of which was then irradiated and Flt-3 administered.^16^ Tumor growth was inhibited on the irradiated but not the un-irradiated side after RT alone. When both RT and Flt-3 were given, delayed growth was observed on both irradiated and non-irradiated sides. TLRs augment presentation of antigen, upregulating costimulatory molecules on dendritic cells and stimulating the production of cytokines. The TLR7 ligand agonist (imiquinod) has been combined with radiation in a mouse model of skin metastases from breast cancer.^49^ Primary and secondary sites were injected with breast cancer cells and tumor growth was suppressed at both sites when imiquinod was applied topically to the primary site alone (reduction of tumor volumes). This inhibitory effect was enhanced if both primary and secondary sites were treated with imiquimob but only the primary site irradiated.

### *Clinical studies of radiation combined with checkpoint inhibitors*

Immune checkpoints play a key role in modulating the inflammatory response to infection. In the tumor microenvironment, disrupted regulation of these immune checkpoints compromises host immune response to cancer. Cytotoxic T lymphocyte-associated protein-4 (CTLA-4) and programmed cell death-1 (PD-1) are two checkpoint inhibitors that have been extensively evaluated. A number of clinical trials are in progress evaluating a combination of CTLA4/PDI/PDL1 in inhibitors in patients with metastatic breast cancer.^50,51^ In a phase 2 study of metastatic triple negative breast cancer the PDL1 Inhibitor pembrolizumab is being combined with radiation (5 × 6 Gy over 5–7 days) for patients with at least two measurable tumors.^52^ In a study combining radiation with GM-CSF and local irradiation of metastatic solid tumors (3.5 Gy × 10), 5 out of 14 breast cancer patients had a demonstrable abscopal response.^18^ Clinical trials combining radiation therapy and immune checkpoint blockade have shown responses in up to 19% of breast cancer patients (some of which are durable).^20^ Any abscopal response may be influenced by the volume of the irradiated tumor. It has been hypothesized that larger tumors may release larger amounts and variety of neoantigens.^53^ The relative infrequency of abscopal effects in response to combined radiation and immune checkpoint inhibitors in breast cancer may partly be due to immunosuppressive regulators within the tumor microenvironment. These include transforming growth factor beta, regulatory T cells and myeloid derived suppressor cells (MDSCs) which collectively have an immunosuppressive role.

Counteracting these immunosuppressive regulators is being explored in preclinical murine models. When a neutralizing antibody to TGF beta was administered in combination with radiation (5 daily doses of 6 Gy) to mice bearing breast tumor xenografts, there was dendritic cell activation and a strong CD8+response. Tumor growth was inhibited in both locally irradiated tumors and distant metastases, although tumor progression occurred after an initial response^54^

Caution is needed when extrapolating from evidence based on different tumor models involving a variety of dose and fractionation regimens. The optimal dose, fraction size, and timing of radiation in conjunction with immunomodulation to maximize an abscopal response remains to be determined and a ‘one size fits all’ approach is unlikely to be applicable. In particular, it is unclear whether a single low dose of radiation (e.g., 0.5–2.5 Gy) more effectively suppresses tumor growth, compared with hypofractionated doses of 6–8 Gy or even standard doses of 2 Gy which may themselves be more efficacious than a large single dose.^20^ It is unusual for conventional fractionated doses of radiotherapy alone to generate an abscopal response, implying that immunostimulatory properties of radiation are inadequate to counteract the immunosuppressive microenviroment in most human tumors.^55^ A meta-analysis of trials of adjuvant regional nodal irradiation has shown improved disease-free, metastases-free and overall survival in stage 1-111A breast cancer.^56^ However, on the basis of pre-clinical studies on breast tumors, it seems unlikely that these improved outcomes can be attributable to an abscopal effect of radiotherapy alone on local treatment of nodal ‘oligometastases’.

Despite promising research, molecular mechanisms underlying the abscopal effect remain incompletely understood and future research efforts should focus on identifying the optimum sequencing of immunotherapy in relation to local radiation treatment. Insights into potential mechanisms for an abscopal effect come from work with local radiotherapy combined with immunotherapy in mice in which an irradiated tumor is converted to an in situ vaccine. A key step in this process is induction of interferon type-1 (IFN-1) which can recruit dendritic cells and prime T-lymphocytes involved in cell-mediated immunity. Irradiation of tumors produces double-stranded DNA fragments which can migrate into the cytoplasm and activate a synthase enzyme (cGAS) and its adapter protein STING, thus triggering IFN-1.^57^ Herrera and colleagues provide a useful framework to address these clinical questions^58^ Three scenarios need to be considered: 1) Immune therapy added to standard hypofractionated radiotherapy oligometastatic disease (aiming to take advantage of the in situ vaccination effect of radiation); 2) Immunotherapy combined with standard chemo-radiation (to enhance effect of chemo-RT on local and systemic synergy of RT and immunomodulation); 3) Radiation added to immunotherapy (aiming to maximize the efficacy of immunotherapy with radiation acting as biological response modifiers). Useful guidelines on immune-related response criteria are available.^59^ The extent to which studies of the abscopal effect of radiotherapy in the metastatic setting can be extrapolated to the adjuvant setting, in which the primary tumor has been resected followed by systemic treatment, remains unknown. However, advanced disease provides the context for understanding mechanisms that might be relevant to adjuvant postoperative radiotherapy (i.e., after mastectomy or breast conserving surgery). The size of nodal deposits may be critical in terms of an abscopal response, with larger tumors generating more immunostimulatory neo-antigens but disproportionate enhancement of the immunosuppressive microenvironment.^53^ Negating these immunosuppressive effects is important, and a strategy of dual blockade may specifically stimulate the immune system and negative immunosuppressive effects of radiotherapy.

### *Biomarkers of immune response*

Responses to immune checkpoint blockade are variable and unpredictable between and within any single tumor type. To harness abscopal effects into clinically meaningful treatments, biomarkers of immune response are needed. DNA damage and repair significantly influence interactions between tumors and the immune system, with DNA repair being an important biomarker of immune checkpoint blockade response.^55^ DNA repair deficiency leads to a greater mutational load and quantity of neo-antigen and levels of both correlate with response rates. However, a high mutational load is not essential for generation of an immune checkpoint blockade response. It is likely that a spectrum of immunological effects is associated with various DNA repair deficient settings.^55^ A greater understanding is needed of the impact of genomic instability which may be induced in non-irradiated tissues via a bystander effect. Clinical validation of DNA as a biomarker is necessary before use in clinical decision-making for selection of patients likely to benefit from combined radiation and immunomodulation.

A recent meta-analysis of abscopal effects in preclinical models (including breast cancer) estimates that a biologically effective dose (BED) of 60 Gy has a 50% probability of inducing an abscopal effect, although the relevance of these findings to breast cancer in women is uncertain.^60^ Despite significant heterogeneity in the dose and fractionation regimens employed in the Oxford overview, it should be noted that a meta-analysis of trials of post-mastectomy radiotherapy used biologically equivalent doses. Trials using a BED of 40–60 Gy with an appropriate target volume were associated with a 6.4% absolute increase in survival up to 10 years (OR of death = 0.78; 95% CI 0.70–0.85; *p* < 0.001).^61^

However, whether there is a dose threshold for the abscopal effect is uncertain. Moreover, we do not know if the standard dose and fractionation regimes of adjuvant radiotherapy are optimal for leveraging the abscopal effect, nor whether radiotherapy administered before breast surgery might augment any abscopal effects.^62^ We need biomarkers of an abscopal effect of radiation which could be tested on clinical datasets of patients treated by mastectomy or breast conserving therapy, and appropriate systemic therapy randomized in clinical trials to postoperative radiotherapy or no radiotherapy. To date, there are no published molecular signatures of the abscopal effect in breast cancer or any other solid tumor and no validated biomarkers of likely response to combined radiotherapy and immunotherapy.^63^ Potential immunological surrogates for immune response are under investigation and include cytokine profiles, antibody titers changes in peripheral blood immune cells, and tumor infiltrating immune cell phenotypes. In addition, we need to know more about the factors which modulate the balance between the immune-activating and immunosuppressive signals induced by radiation, and the targets which would allow radiation immunity to be enhanced.^64^

If our hypothesis is validated by further data, the impact on clinical practice could be transformational. We might in future be able to identify at diagnosis subsets of patients whose breast tumors have the capacity for an immune-mediated abscopal effect from an optimal dose fractionation regime enacted and enhanced by specific adjuvant systemic therapies. The purpose of radiotherapy would shift from direct tumor cell kill to modulation of the immune system consequent to increased neo-antigen exposure. Out-of-target metastatic tumor foci would therefore be targeted not only with conventional systemic therapies, but also these distant bystander immune effects, thus increasing the chance of eradicating distant disease foci.

## Conclusion

Potential abscopal effects following radiotherapy should be exploited to further lower breast cancer mortality. New immunotherapeutic agents such as checkpoint inhibitors and TLR agonizts could be developed to enhance this abscopal effect and thereby produce a more robust response. Moreover, use of dual immune checkpoint blockade may help prevent adaptive immune resistance and maintain the pro-immunogenic effects of local radiotherapy.^65^ The potential synergism between chemotherapy and breast cancer radiotherapy might be bolstered by future development of similar (but less toxic) immunogenic modulators to maximize the abscopal response. It should be noted that systemic therapies have largely been effective in reducing the risk of breast cancer recurrence and deaths during the initial three to five years following diagnosis.^10^ With increasing numbers of long-term breast cancer survivors, there is an urgent need to lower the burden of delayed recurrences and deaths. Harnessing the abscopal response within the context of a permissive host/tumor environment may provide an opportunity to do so. A better biological understanding of how abscopal effects are dependent on factors such as radiation dose, fractionation, and timing will help maximize the clinical impact and may serve to further lower the global burden of breast cancer mortality. Current consensus is that multidisciplinary collaboration will permit leverage of abscopal effects which could transform the field of radiation oncology.^19^ This would apply to breast cancer, as well as other solid tumors in which radiotherapy has a key role in curative therapy. Further research will be needed to confirm or refute this hypothesis.
